# Domperidone Protects Cells from Intoxication with *Clostridioides difficile* Toxins by Inhibiting Hsp70-Assisted Membrane Translocation

**DOI:** 10.3390/toxins15060384

**Published:** 2023-06-07

**Authors:** Maria Braune-Yan, Jinfang Jia, Mary Wahba, Johannes Schmid, Panagiotis Papatheodorou, Holger Barth, Katharina Ernst

**Affiliations:** Institute of Experimental and Clinical Pharmacology, Toxicology and Pharmacology of Natural Products, Ulm University Medical Center, 89081 Ulm, Germany

**Keywords:** toxin uptake, chaperones, Hsp70, domperidone, *Clostridioides difficile* toxins, toxin inhibitors

## Abstract

*Clostridioides difficile* infections cause severe symptoms ranging from diarrhea to pseudomembranous colitis due to the secretion of AB-toxins, TcdA and TcdB. Both toxins are taken up into cells through receptor-mediated endocytosis, autoproteolytic processing and translocation of their enzyme domains from acidified endosomes into the cytosol. The enzyme domains glucosylate small GTPases such as Rac1, thereby inhibiting processes such as actin cytoskeleton regulation. Here, we demonstrate that specific pharmacological inhibition of Hsp70 activity protected cells from TcdB intoxication. In particular, the established inhibitor VER-155008 and the antiemetic drug domperidone, which was found to be an Hsp70 inhibitor, reduced the number of cells with TcdB-induced intoxication morphology in HeLa, Vero and intestinal CaCo-2 cells. These drugs also decreased the intracellular glucosylation of Rac1 by TcdB. Domperidone did not inhibit TcdB binding to cells or enzymatic activity but did prevent membrane translocation of TcdB’s glucosyltransferase domain into the cytosol. Domperidone also protected cells from intoxication with TcdA as well as CDT toxin produced by hypervirulent strains of *Clostridioides difficile*. Our results reveal Hsp70 requirement as a new aspect of the cellular uptake mechanism of TcdB and identified Hsp70 as a novel drug target for potential therapeutic strategies required to combat severe *Clostridioides difficile* infections.

## 1. Introduction

*Clostridioides (C.) difficile*, formerly known as *Clostridium difficile*, is a spore-forming bacterium that can cause severe gastrointestinal infections in humans. These *C. difficile* infections (CDI) cause mild to severe diarrhea that can develop into life-threatening conditions such as pseudomembranous colitis, colonic perforation or toxic megacolon [1]. CDI is one of the most common causes of healthcare associated diarrhea and is linked to more than 200,000 hospitalizations in the US alone, leading to a significant healthcare burden, causing morbidity, mortality and increased healthcare costs [2]. Infections mainly occur if the gut microbiome has been disrupted, typically by previous antibiotic treatment. This allows *C. difficile* spores to develop to their vegetative form and then grow and secrete highly potent AB-type protein toxins, toxin A (TcdA) and toxin B (TcdB), which are the causative agents and main virulence factors for CDI symptoms [3]. TcdA and TcdB are encoded by the tcdA and tcdB genes, respectively, and are expressed in response to environmental cues such as exposure to bile acids [4]. Hypervirulent strains of *C. difficile* have distinct characteristics compared to other strains. They produce higher levels of TcdA and TcdB and exhibit greater resistance to antibiotics such as fluoroquinolones. In addition, these strains also produce a third toxin, the *C. difficile* transferase CDT, an actin ADP-ribosylating toxin that leads to the destruction of the actin cytoskeleton. Hypervirulent strains are associated with more severe disease, higher recurrence rates and enhanced mortality [3,5].

TcdA and TcdB are large (~300 kDa) glucosylating toxins that share about 50% sequence identity and act as AB-type toxins in the cytosol of target cells. Due to their complex structure, TcdA and TcdB are further classified as ABCD-type toxins. They consist of four functional domains: an enzymatically active glucosyltransferase domain (GTD) at the N-terminus (A-domain), an autoprotease domain (APD) (C-domain), a translocation domain that forms pores into endosomal compartments (D-domain) and a binding domain for receptor-binding of the toxin at the cell surface (B-domain). After receptor-binding, the toxins are internalized by receptor-mediated endocytosis. The acidification of endosomes leads to conformational changes in the toxins resulting in the formation of a translocation pore into the endosomal membrane [6]. The APD and GTD are translocated through this pore to the cytosol [7,8]. Cytosolic inositol hexakisphosphate (InsP_6_) triggers the autoproteolytic activity of the APD leading to release of the GTD from the holotoxin [9,10]. The GTD glucosylates GTPases of the Rho-/Ras-family such as Cdc42, Rho or Rac1, which are located at the cell membrane [11,12]. They regulate numerous important functions and processes of cells such as cell migration, epithelial barrier integrity and cytokine production and are therefore referred to as molecular switches. Glucosylation inactivates these GTPases leading to release of certain cytokines and destruction of the cytoskeleton which in turn results in rounding of cells, loss of cell–cell contacts, cell cycle arrest and cell death [13]. In vivo, the inactivation of GTPases by the toxins is the major cause for gut barrier disruption, intestinal damage and appearance of clinical symptoms [2].

The treatment of CDI involves specific antibiotics including metronidazole, vancomycin and fidaxomicin, with the last two being superior to metronidazole. A particular problem is recurrent CDI, which is difficult to combat, and the risk of multiple relapses increases significantly with the first recurrence [2,14]. Since *C. difficile* toxins have been identified as one risk factor for recurrence, toxin-directed strategies take on greater significance. Bezlotoxumab, a human monoclonal antibody against TcdB, is in clinical use to reduce the risk of CDI recurrence [15].

In recent years, the role of host cell factors such as heat shock proteins and other chaperones during the translocation of bacterial AB-type toxins has been characterized (for a review, see [16]). Amongst others, clostridial binary toxins such as *Clostridium botulinum* C2 toxin and *C. difficile* CDT or *Bordetella pertussis* toxin and *Vibrio cholera* toxin require the activity of Hsp90, Hsp70, cyclophilins (Cyps) or FK506-binding proteins (FKBPs) for uptake of their enzyme subunits into the cytosol of target cells [17,18,19,20,21,22,23,24,25,26,27,28,29,30]. Interestingly, the intoxication of cells with TcdA or TcdB was independent of Hsp90, Cyps and FKBPs [17,19,25,31,32]. In the present study, we uncovered that Hsp70 activity is required for translocation of the GTD of TcdB into the cytosol and therefore for successful intoxication of cells with TcdB.

For characterizing the role of Hsp70 during TcdB uptake, we used specific pharmacological inhibitors of Hsp70 activity. VER-155008 (VER) is an established Hsp70 inhibitor that binds to the ATP binding site of Hsp70 [33]. ATP-binding and -hydrolysis is crucial for the function of Hsp70 during protein folding, translocation of proteins across membranes or refolding of misfolded proteins [34]. VER was previously used to characterize the role of Hsp70 during the uptake of other AB-type toxins such as pertussis toxin, diphtheria toxin or cholera toxin [24,26,35]. Recently, domperidone was discovered as a novel inhibitor of Hsp70 activity by a bioinformatics search for FDA-approved drugs that are structurally similar to the allosteric Hsp70 inhibitor HS-72, which also affects the ATP-binding site of Hsp70 [36]. Since the treatment of CDI is still challenging and novel therapeutic strategies focusing on the targeted pharmacological inhibition of the activity of the disease-causing toxins must be extended, we investigated the potential of the licensed drug domperidone (Dom) to protect human cells from TcdB intoxication and characterized the underlying inhibitory mechanism.

## 2. Results

### 2.1. Pharmacological Inhibition of Hsp70 Reduces the TcdB Induced Cell Rounding in a Time- and Concentration-Dependent Manner

**Figure 1:**
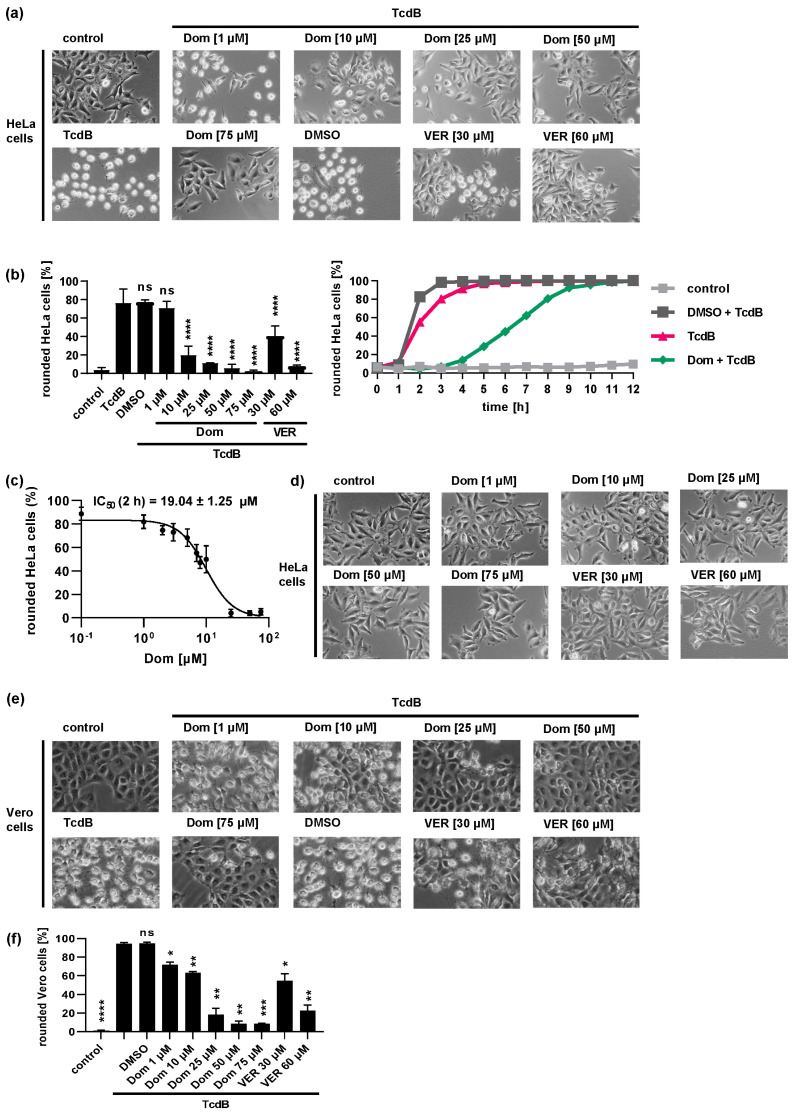
Hsp70 inhibition protects cells from TcdB intoxication ((**a**–**d**) HeLa cells; (**e**,**f**) Vero cells). (**a**) HeLa cells were pre-incubated with domperidone (Dom) or VER for 30 min at 37 °C. For control, cells were treated with DMSO as solvent control or left untreated. TcdB (30 pM) was added, or cells were left untreated (control). Images were obtained after 2 h of intoxication. (**b**) The percentage of rounded cells was determined from cell images shown in (**a**). Data after 2 h of intoxication (left, mean ± SD, n ≥ 3 of one representative of 3 independent experiments) and a time course of intoxication (right) are shown. For the time course, cells were pre-treated with 50 µM Dom and intoxicated with 30 pM TcdB. Values are given as percentage of control; single values per sample of 1 representative of 5 independent experiments are shown. Significance was tested by one-way ANOVA followed by Dunnett’s multiple comparison test. All samples were compared to samples treated with TcdB only. (**c**) The concentration-dependent inhibition of TcdB (30 pM) was investigated by incubating HeLa cells with a concentrations series of Dom ranging from 0.1 µM to 75 µM. After 2 h, images were taken, and percentage of rounded cells was determined. Values are given as mean ± SD (n ≥ 3 values of one representative of 3 independent experiments). Nonlinear fit was applied with GraphPad Prism software via log(inhibitor) vs. response (variable slope, four parameters). Under these conditions, an estimated IC_50_ value of 19.04 ± 1.25 µM was calculated. (**d**) HeLa cells were treated with Dom or VER as described in (**a**). No toxin was added. Images were obtained after 2.5 h of inhibitor treatment. (**e**) Vero cells were treated with Dom, VER or DMSO or left untreated for control for 30 min. TcdB (30 pM) was added, or cells were left untreated (control). Images were obtained after 2 h of intoxication. (**f**) Percentage of rounded cells was determined from cell images shown in (**e**). Values are given as mean ± SD, n ≥ 3 (results of one representative of 3 independent experiments are shown). Significance was tested by one-way mixed-effects analysis followed by Dunnett’s multiple comparison test. All samples were compared to samples treated with TcdB only (**** *p* < 0.0001, *** *p* < 0.001, ** *p* < 0.01, * *p* < 0.05, ns = not significant *p* > 0.05).

**Figure 2:**
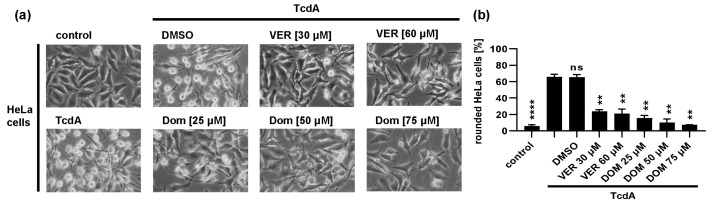
Dom and VER inhibit intoxication of HeLa cells with TcdA. (**a**) HeLa cells were pre-incubated with domperidone (Dom) or VER for 30 min at 37 °C. For control, cells were treated with DMSO as solvent control or left untreated. TcdA (10 nM) was added, or cells were left untreated (control). Images were obtained after 8 h of intoxication. (**b**) Percentage of rounded cells was determined from cell images shown in (**a**). Values are given as mean ± SD, n ≥ 3 (results of one of 3 independent experiments are shown). Significance was tested by mixed-effects analysis followed by Dunnett’s multiple comparison test. All samples were compared to samples treated with TcdA only. (**** *p* < 0.0001, ** *p* < 0.01, ns = not significant *p* > 0.05).

Both Hsp70 inhibitors, VER and Dom, markedly delayed the intoxication of cells with TcdB in a concentration-dependent manner (Figure 1). The rounding of adherent cells such as HeLa or Vero cells results from the intracellular modification of Rho proteins by TcdB. Therefore, cell rounding represents a highly specific endpoint of intoxication indicating cytosolic uptake of the enzyme domain of TcdB. Figure 1a depicts that after 2 h of intoxication, HeLa cells treated solely with TcdB display a high number of rounded cells. If cells were treated with Dom or VER prior to TcdB intoxication, the number of rounded cells was reduced in a concentration-dependent manner. Quantification of rounded cells confirmed the inhibitory effect of VER and Dom on TcdB intoxication after 2 h of TcdB treatment (Figure 1b left). The delay of TcdB intoxication was visible for up to 8 h after intoxication (Figure 1b right). An IC_50_ value for inhibition of TcdB by Dom of approximately 19 µM was determined (Figure 1c). The treatment of cells with Dom alone only showed a slight adverse effect in the highest concentration of 75 µM compared to untreated control samples (Figure 1d). Therefore, a maximal concentration of 50 µM of Dom was used in most of the following experiments. VER and Dom also protected Vero cells from TcdB intoxication (Figure 1e,f) and inhibited intoxication with TcdA (Figure 2).

**Figure 3:**
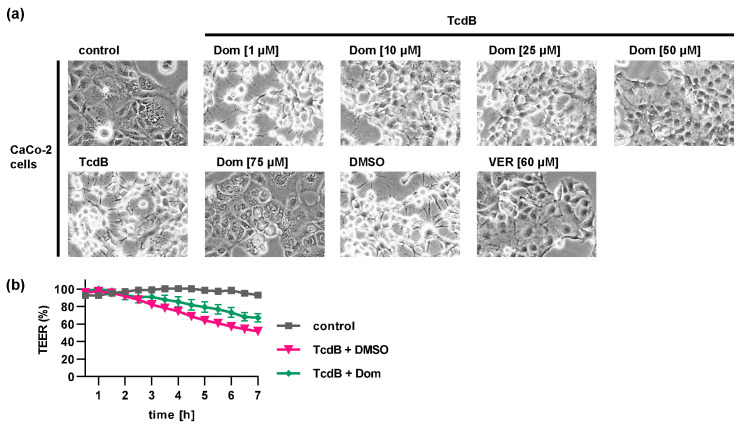
Hsp70 inhibition reduces the intoxication of CaCo-2 cells with TcdB. (**a**) CaCo-2 cells were pre-incubated with domperidone (Dom) or VER for 30 min at 37 °C. For control, cells were treated with DMSO as solvent control or left untreated. TcdB (30 pM) was added, or cells were left untreated (control). Images were obtained after 2 h of intoxication. (**b**) CaCo-2 cells were seeded in culture inserts and treated with 50 µM Dom or DMSO for 30 min from the basolateral side. Then, TcdB (100 pM) was added apically, or cells were left untreated (control). TEER was measured every 30 min. Data were normalized to 0 h time point and are given as mean ± SEM, n ≥ 8 (from 6 independent experiments).

The protective effect of Dom and VER on TcdB intoxication was also shown in the pathophysiologically more relevant human colon carcinoma cell line CaCo-2 (Figure 3a). CaCo-2 cells grow as a tight monolayer on transwell filters and serve as a physiologically relevant model of the intestinal epithelial barrier. TcdB causes a disruption of the epithelial integrity resulting in a decrease in transepithelial electrical resistance (TEER) (Figure 3b). In the presence of Dom, this TcdB-mediated decrease in resistance was delayed.

### 2.2. Pharmacological Inhibition of Hsp70 Leads to Reduced Glucosylation of Intracellular Rac1 by TcdB

**Figure 4:**
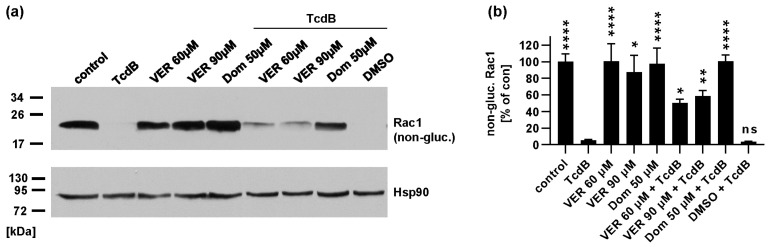
Domperidone inhibits TcdB-mediated glucosylation of intracellular Rac1. HeLa cells were pre-incubated with domperidone (Dom) or VER for 30 min at 37 °C. For control, cells were treated with DMSO as solvent control or left untreated. TcdB (30 pM) was added, or cells were left untreated (control). After 2 h, cells were harvested. Lysates were analyzed by SDS-PAGE and Western blotting. Non-glucosylated Rac1 signal as well as Hsp90 signal for loading control were detected. (**a**) A representative Western blot is shown. (**b**) Western blot signals were quantified and normalized to Hsp90 signals as well as to untreated control samples. Values are given as mean ± SEM (n ≥ 4, of at least 2 independent experiments). Significance was tested by one-way ANOVA analysis and multiple comparisons test and refers to samples treated with only TcdB. (**** *p* < 0.0001, ** *p* < 0.01, * *p* < 0.05, ns = not significant *p* > 0.05).

To confirm the inhibitory effect of VER and Dom against TcdB, the glucosylation status of intracellular Rac1 was determined as an additional endpoint. After cells were intoxicated with TcdB in the presence or absence of different Dom/VER concentrations, lysates of these cells were analyzed by Western blot. A specific antibody was used that only recognizes the unmodified, i.e., non-glucosylated Rac1. Figure 4 shows that lysates from untreated control samples display a strong signal indicating that Rac1 was not glucosylated. Inhibitors alone had no significant effect on detection of unmodified Rac1. In cells treated with TcdB, no unmodified Rac1 was detectable, showing that most of the Rac1 was glucosylated in these cells. In the presence of Dom, a signal for non-glucosylated Rac1 was visible that was stronger than signals from samples treated with TcdB only (Figure 4). VER also inhibited Rac1 glucosylation by TcdB in higher concentrations, but the inhibitory effect of Dom was more pronounced. Therefore, we focused on characterizing the inhibitory effect of Dom in the following experiments.

### 2.3. Domperidone Has no Inhibitory Effect on TcdB Receptor Binding, Autoprotease Activity or In Vitro Glucosyltransferase Activity

**Figure 5:**
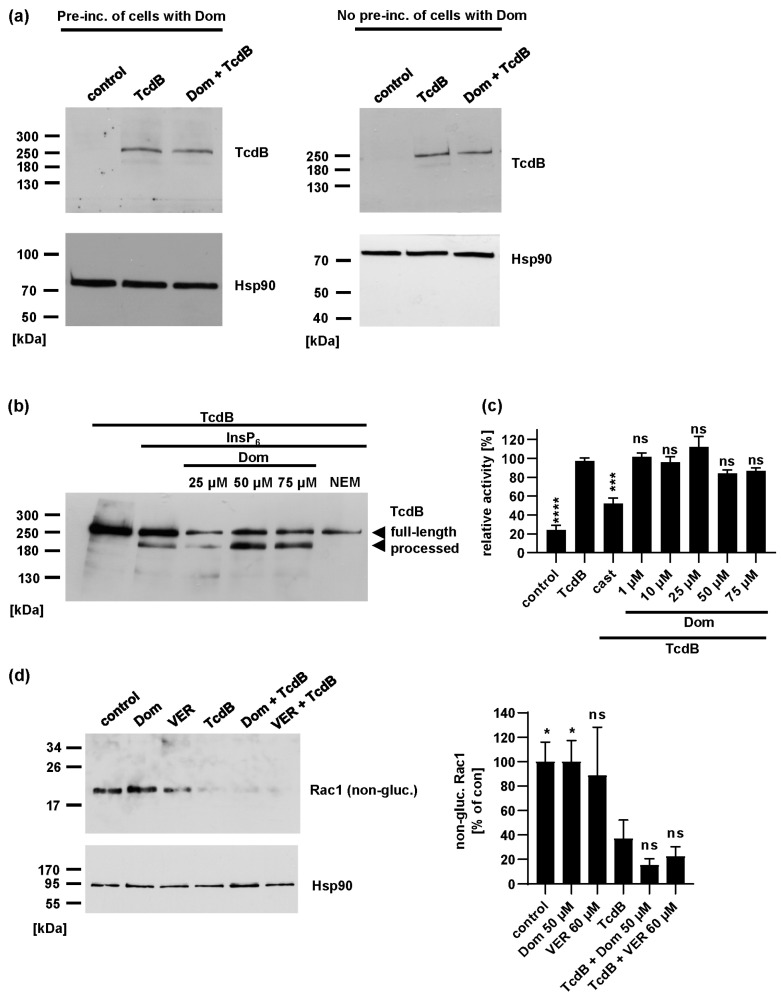
Domperidone has no inhibitory effect on receptor binding, autoproteolysis or enzyme activity of TcdB. (**a**) Vero cells were treated with 50 µM domperidone (Dom), either with (left) or without (right) 30 min pre-incubation at 37 °C, before TcdB (500 pM) was added for 1 h on ice. TcdB and Hsp90 (loading control) were detected by Western blotting using specific antibodies. pre-inc. = pre-incubation (**b**) For assessing the effect of Dom on the autoproteolytic activity of TcdB, 2 µg TcdB was incubated with indicated concentrations of Dom plus inositol hexakisphosphate (InsP_6_). For control, TcdB was treated with InsP_6_ alone or left untreated. N-ethylmaleimide (NEM) was included as a known inhibitor of TcdB autoproteolytic activity. Samples were incubated for 1 h at 37 °C and subsequently subjected to SDS-PAGE and Western blotting. Full-length TcdB and processed fragments were detected using a specific antibody. (**c**) UDP-Glo^TM^ glucosyltransferase assay was performed to determine the effect of Dom on TcdB glucosyltransferase activity. A total of 200 pM TcdB were incubated with indicated concentrations of Dom in the presence of Rac1. Castanospermin (cast) was included as a known inhibitor of TcdB glucosyltransferase activity. Via the addition of UDP-glucose, the reaction was initiated and proceeded for 1 h at 37 °C. The UDP detection reagent was added, and luminescence was measured. Values were normalized to samples treated only with TcdB and are given as mean ± SEM, n ≥ 13 values from 5 independent experiments. Outliers were removed according to GraphPad Prism outlier test. Significance was tested by mixed-effects analysis and multiple comparisons test and refer to samples treated only with TcdB. (**d**) 40 µg cell lysate was incubated with 50 ng TcdB with or without Dom or VER for 2 h at 37 °C. For control, lysate was incubated with VER or Dom alone. Non-glucosylated Rac1 was detected by Western blot analysis. A representative blot (left) and quantification of Western blot signals (right) are shown. Quantified signals were normalized to Hsp90 signals and to untreated control. Values are given as mean ± SEM, n ≥ 6 values from 4 independent experiments. Significance was tested against samples treated only with TcdB by mixed-effects analysis and multiple comparisons test. (**** *p* < 0.0001, *** *p* < 0.001, * *p* < 0.05, ns = not significant *p* > 0.05).

To unravel the underlying mechanism of inhibition, it was next tested whether Dom inhibits crucial steps of TcdB uptake and its mode of action. Dom had no inhibitory effect on binding of TcdB to cells (Figure 5a). For releasing the enzyme domain of TcdB into the cytosol, the autoproteolytic activity is required. The incubation of TcdB with InsP_6_ triggers autoproteolysis. Figure 5b shows that in the presence of different Dom concentrations, TcdB is still autoproteolytically processed. For control, N-ethylmaleimide (NEM) was included as a known inhibitor of autoproteolysis [10]. Glucosyltransferase activity of TcdB in vitro was not inhibited by Dom, as demonstrated by the UDP-Glo^TM^ glucosyltransferase assay (Figure 5c) and by the detection of non-glucosylated Rac1 via Western blot (Figure 5d).

### 2.4. Domperidone Inhibits pH-Driven Membrane Translocation of the Enzyme Domain of TcdB

**Figure 6:**
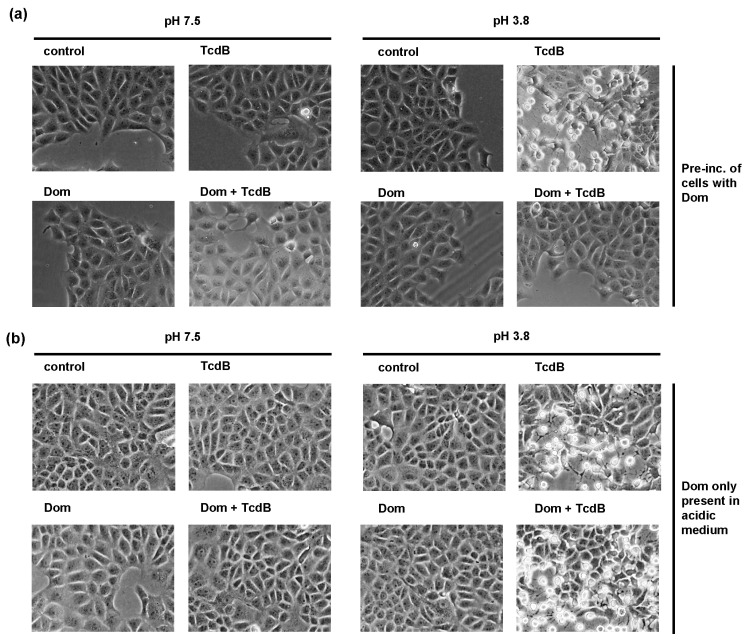
Domperidone inhibits membrane translocation of TcdB. (**a**) Vero cells were pre-incubated with domperidone (Dom, 50 µM) for 30 min or left untreated. All samples were treated with bafilomycinA1 (BafA1, 100 nM) for another 30 min. Cells were incubated on ice for 10 min, followed by addition of TcdB (500 pM) for 30 min on ice. For control, cells were left untreated (control). Then, warm acidic medium containing 50 µM Dom was added for 10 min at 37 °C. For control, warm neutral medium with 50 µM Dom was added. Subsequently, medium was removed and replaced with BafA1-containing medium. Morphological changes were monitored, and images show cells after 6 h of intoxication. (**b**) Vero cells were incubated with BafA1 (100 nM) for 30 min. Then, cells were put on ice for 10 min, followed by addition of TcdB (500 pM) for 30 min on ice. For control, cells were left untreated (control). Then, warm acidic medium containing 50 µM Dom was added for 10 min at 37 °C. For control, warm neutral medium with 50 µM Dom was added. Afterwards, medium was replaced with BafA1-containing medium, and morphological changes were monitored. Images show cells after 5 h of intoxication. Pre-inc. = pre-incubation.

Since Dom had no effect on the receptor binding, autoproteolytic activity and enzyme activity, another step of TcdB uptake must be affected by Dom. It was already shown before that Hsp70 facilitates the translocation of other AB-type toxins across endosomal membranes. Therefore, we next investigated if Dom inhibits TcdB membrane translocation in an isolated manner. To achieve this, we aimed to replicate the acidic conditions found in endosomes directly at the cytoplasmic membrane as described before [8]. By doing so, we could induce translocation of the enzyme domain across the cytoplasmic membrane without relying on the endocytosis route and subsequent translocation from endosomes. To initiate this process, cells were incubated with TcdB while kept on ice to facilitate binding while prevent internalization. Challenging cells with warm acidic medium triggers pore formation by TcdB directly into the cytoplasmic membrane and translocation of the glucosyltransferase domain into the cytosol. To block the acidification of endosomes and therefore the physiological uptake route of TcdB, cells were also treated with bafilomycinA1 (BafA1). BafA1 inhibits the vesicular ATPase which pumps protons into the endosome. Pre-incubation of cells with Dom prevented membrane translocation of TcdB (Figure 6a). If Dom was only present during the acidification step, inhibition was not observed (Figure 6b).

### 2.5. Domperidone Reduces Intoxication of Cells with Binary CDT

**Figure 7:**
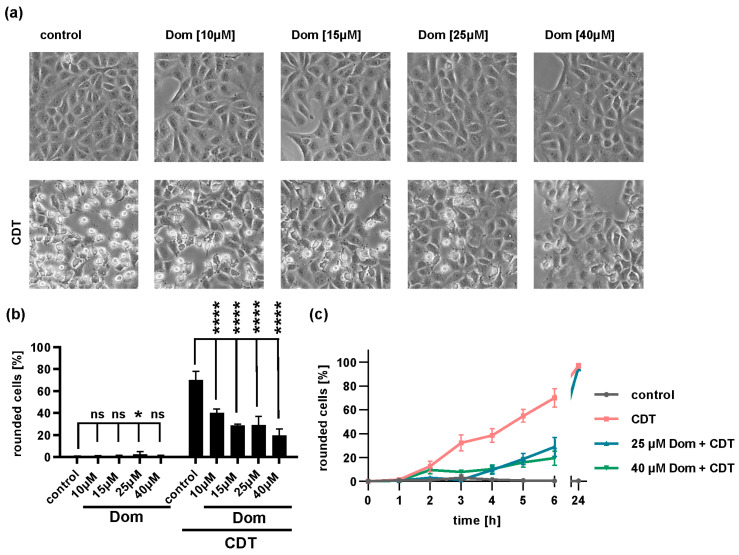
Domperidone protects cells from CDT intoxication. Vero cells were pre-incubated with indicated concentrations of domperidone (Dom) for 30 min at 37 °C or left untreated for control. Then, cells were challenged with CDT (150 ng/mL CDTa plus 300 ng/mL CDTb), or cells were further incubated with the inhibitor alone. Pictures were taken every hour. (**a**) Morphological changes after 6 h of CDT intoxication are shown. (**b**) The percentage of rounded cells was determined after 6 h of CDT intoxication from images. Values are given as mean ± SD, n = 3 values of one representative experiment. Significance was tested by one-way ANOVA and Dunnett’s multiple comparisons test. (**c**) A time-course of intoxication with CDT is shown. Values are given as mean ± SD, n = 3 values of one representative experiment. (**** *p* < 0.0001, * *p* < 0.05, ns = not significant *p* > 0.05).

Hypervirulent *C. difficile* strains produce the ADP-ribosylating CDT toxin in addition to TcdB and TcdA. CDT ADP-ribosylates G-actin leading to depolymerization of F-actin and rounding of adherent cells (Figure 7). Cells treated with increasing concentrations of Dom and CDT showed fewer rounded cells compared to cells treated with CDT alone indicating inhibition of intoxication (Figure 7a). Quantification of rounded cells confirmed the inhibitory effect of Dom on CDT intoxication of cells (Figure 7b,c).

Taken together, the results indicate that Hsp70 inhibitors Dom and VER reduced the intoxication of cells with TcdB by analyzing TcdB-induced cell morphology as well as the glucosylation status of intracellular TcdB substrate Rac1 and TcdB-mediated decrease in resistance of CaCo-2 monolayers. Dom had no effect on TcdB cell binding, autoprotease activity or enzyme activity in vitro but inhibited membrane translocation of the glucosyltransferase domain of TcdB into the cytosol of target cells. Moreover, the intoxication of cells with TcdA as well as the binary CDT toxin, produced by hypervirulent strains in addition to glucosylating toxins, was also reduced by Dom.

## 3. Discussion

Hsp70 is a highly conserved, abundant protein that is part of the molecular chaperone family and works as a co-chaperone of Hsp90. It plays a role in various cellular processes, including protein folding, translocation of polypeptide chains into the mitochondria or endoplasmic reticulum, and preventing protein aggregation and misfolding. Hsp70 helps in protein translocation by binding the protein close to the translocation pore and generating a one-way pulling motion, known as entropic pulling [34,37,38]. Hsp70 comprises four domains: the nucleotide binding domain, substrate binding domain, helical lid domain and C-terminal tail domain [34]. ATP binding and hydrolysis are vital for Hsp70 function, and inhibitors such as VER-155008 (VER) prevent this process [33].

The requirement of Hsp70 for membrane translocation has been shown for other AB-type toxins before (for review see [16]). ADP-ribosylating toxins including clostridial binary toxins C2 toxin, iota toxin and CDT toxin [21,22], diphtheria toxin [35], pertussis toxin [24] and cholera toxin [26] depend on Hsp70 activity for the uptake of their enzyme subunits into the cytosol of host cells and therefore for intoxication of cells. These toxins also rely on the activity of additional cellular chaperones, namely, Hsp90, cyclophilins (Cyps) and FK506 binding proteins (FKBPs) [16]. During these investigations, it was shown that the uptake of TcdB occurred without dependence on Hsp90, while the uptake of TcdA occurred without reliance on Cyps and FKBPs [17,19,25]. Given the close structural and functional relationship between TcdA and TcdB, it is highly probable that both toxins operate independently of Hsp90, Cyps and FKBPs but require Hsp70 activity. These findings point towards a common necessity for chaperones in uptake of AB-type toxins. However, it is notable that the specific types of chaperones required can differ depending on the toxin in question.

Later, it was shown that TcdB and TcdA need the assistance of the chaperonin TRiC/CCT (TCP-1 ring complex, chaperonin containing TCP-1) for efficient refolding and restoration of the glucosyltransferase activity of the enzyme domain [32]. TRiC/CCT is a ring-shaped molecular machine involved in the folding of many newly synthesized proteins. It appears that approximately 10% of cytosolic proteins interact with TRiC/CCT [39,40]. While the CCT4/5 subunits of TRiC/CCT have been shown to be effective in refolding of the TcdB enzyme domain (TcdB^GT^), it is not known if they are involved in membrane translocation [32]. Notably, the enzyme activity of TcdB^GT^ was not recovered by Hsp90, and only a weak recovery effect was seen with Hsp70 [32]. However, our findings clearly demonstrate that Dom effectively inhibits membrane translocation of TcdB, suggesting that TRiC/CCT and Hsp70 perform complementary functions. Hsp70 aids in the translocation from the endosome to the cytosol, possibly through entropic pulling that has been described for Hsp70 before, while TRiC/CCT takes over refolding and restoration of TcdB^GT^ enzyme activity.

Recent research has revealed that Dom has an inhibitory effect on Hsp70 by screening a library of FDA-approved drugs for compounds with structural similarity to the known Hsp70 inhibitor HS-72 [36,41]. In a study conducted by Concilli et al. in 2020, it was shown that Dom and HS-72 both significantly reduced the ATPase activity of Hsp70, with Dom exhibiting an even stronger effect than HS-72 [36]. Dom is widely used in many countries to treat nausea and vomiting associated with conditions such as gastroesophageal reflux disease (GERD) or gastroparesis. Adverse effects include headache, dry mouth, dizziness, and gastrointestinal disturbances. In rare cases, serious adverse effects such as cardiac arrhythmias were reported [42,43].

The treatment of CDI typically involves the use of specific antibiotics that target the bacteria causing the infection, such as metronidazole, vancomycin and fidaxomicin [1]. However, treatment is particularly challenging in cases of recurrent CDI. Bezlotoxumab is a monoclonal antibody approved by the FDA in 2016 for use in combination with standard antibiotics for the treatment of CDI in adults who are at high risk of recurrence [44]. It works by binding to and specifically neutralizing TcdB, which can cause severe inflammation and damage to the intestinal lining. In combination with standard antibiotic therapy, bezlotoxumab reduces the rate of CDI recurrence. Therefore, bezlotoxumab is considered an effective treatment option for reducing the risk of recurrence in patients with *C. difficile* infection [15,44].

Bezlotoxumab is the only approved drug targeting a *C. difficile* toxin and only neutralizes TcdB that has not yet bound to cells [44]. Since TcdA and TcdB cause the severe symptoms during the infection, new therapeutic strategies should focus on targeting these toxins. In this regard, inhibiting Hsp70 to prevent translocation of the enzyme domain into the cytosol represents an opportunity to neutralize toxin molecules that have already been internalized. Domperidone, as an approved drug with a known safety profile, may offer an option to extend the therapeutic approach to treat CDI. However, careful consideration of therapeutic efficacy versus adverse effects, depending on the severity of the disease, is necessary.

Hypervirulent strains of *C. difficile* are associated with more severe disease, higher rates of recurrence and increased mortality. They produce the binary CDT toxin in addition to TcdA and TcdB. Previous studies have shown that the ADP-ribosylating CDT toxin also requires Hsp70 as well as Hsp90, Cyp and FKBP activity for membrane translocation of its enzyme component into the cytosol, making Dom a potentially interesting treatment option for infections caused by hypervirulent *C. difficile* strains [21,22,45,46]. Additionally, other toxins such as pertussis toxin, diphtheria toxin and cholera toxin, which also cause severe diseases, depend on Hsp70 for their action. Therefore, Dom treatment might have therapeutic potential for treatment of diseases caused by these bacterial toxins, including whooping cough, diphtheria or cholera.

## 4. Materials and Methods

### 4.1. Protein Expression and Purification

Toxin components CDTa and CDTb were expressed, purified and activated as recombinant proteins as described before [47]. Native TcdB from *C. difficile* VPI 10,463 was expressed and purified as described earlier [48]. Rac1 was expressed and purified as a recombinant GST-tagged protein as described previously [49]. Native TcdA was obtained from List (Campbell, CA, USA).

### 4.2. Cell Culture and Intoxication Experiments

HeLa (DSMZ, Braunschweig, Germany) and Vero cells (African green monkey kidney cells, DSMZ, Braunschweig, Germany) were cultivated in minimum essential medium (MEM) with 10% heat-inactivated fetal calf serum (FCS) from GIBCO life technologies (Karlsruhe, Germany), 1 mM sodium pyruvate (Thermo Fisher Scientific, Waltham, MA, USA), 2 mM L-glutamine (PAN-Biotech, Aidenbach, Germany), 0.1 mM non-essential amino acids, 100 U/mL of penicillin and 100 µg/mL of streptomycin (Thermo Fisher Scientific).

CaCo-2 cells (human epithelial colorectal adenocarcinoma cells, ATCC HTB-37, Manassas, VA, USA) were cultured in Dulbecco’s Modified Eagle Medium (DMEM) from GIBCO life technologies with 10% FCS, 1 mM sodium pyruvate, 0.1 mM non-essential amino acids, 100 U/mL penicillin and 100 µg/mL streptomycin.

Humidified conditions with 5% CO_2_ at 37 °C were applied to incubate cells. For intoxication experiments, cells were detached with trypsin and seeded into 24-well plates (TPP Techno Plastic Products, Trasadingen, Switzerland). Medium was exchanged, and cells were pre-incubated with domperidone (Sigma Aldrich by Merck, Darmstadt, Germany), VER (inhibitor of ATP- binding site of Hsp70, Hsc70 and Grp78, Tocris Bioscience, Wiesbaden-Nordenstadt, Germany), BafA1 (Calbiochem, Bad Soden, Germany), DMSO as solvent control or medium only for 30 min. Then, toxin was added, and cell images were taken after different time points using a Zeiss (Oberkochen, Germany) Axiovert 40CFL microscope with a Jenoptik (Jena, Germany) ProGres C10 CCD camera/Leica DMi1 microscope connected to a Leica MC170 HD camera (both Leica Microsystems GmbH, Wetzlar, Germany). The percentage of rounded, i.e., intoxicated, cells was determined from images using neuralab.de.

To obtain a time course of intoxication, the zenCELL owl 24 channel automated microscope (innoME GmbH, Espelkamp, Germany) was used. Images were obtained every 10 min for 8–12 h.

### 4.3. Glucosylation Status of Intracellular Rac1 after TcdB-Treatment

Cells were pre-incubated with the respective inhibitors and treated with TcdB as described above. After 2 h, cells were washed and lysed by freezing. Cell lysates here harvested in 2.5 Laemmli + DTT. Samples were subjected to SDS-PAGE followed by Western blotting. Non-glucosylated Rac1 (1:1000, #610651, BD Biosciences, Franklin Lakes, NJ, USA) as well as Hsp90 (1:1000, Santa Cruz Biotechnology, Heidelberg, Germany) were detected by specific primary antibodies and horseradish peroxidase-coupled secondary antibodies (Santa Cruz Biotechnology) using the enhanced chemiluminescence system (ECL, Millipore, Merck, Darmstadt, Germany). Densitometric quantification of Western blot signals was determined using ImageJ (v1.53p, U.S. National Institutes of Health, Bethesda, MD, USA). Values were normalized to loading control signals (Hsp90).

### 4.4. Cell-Binding Assay

Cells were treated with Dom, either with or without a 30 min pre-incubation. Then, cells were transferred to ice for 10 min before TcdB was added for 1 h on ice. Samples were subjected to SDS-PAGE and Western blotting. TcdB (1:1000, MCA4737, BioRad, Hercules, CA, USA) and Hsp90 were detected by specific antibodies.

### 4.5. In Vitro Autoprocessing of TcdB

Effects of Dom on the intrinsic cysteine protease activity of TcdB were tested by incubating TcdB with or without different concentrations of Dom for 1 h at 37 °C in a 20 mM Tris-HCl buffer containing 150 mM NaCl at pH 7.4. To induce autoprocessing activity, 1 mM inositol hexakisphosphate (Santa Cruz Biotechnology) was added. As a positive control for inhibition of autoprocessing, 1 mM N-ethylmaleimide (Sigma Aldrich by Merck) was used. To stop the reaction, Laemmli buffer was added, and samples were denatured at 95 °C. Samples were subjected to SDS-PAGE (8% gel) followed by Western blotting. TcdB and fragments were detected by a specific primary antibody (1:1000, MCA4737, BioRad).

### 4.6. UDP-Glo^TM^ Glucosylation Assay

The assay was performed according to the manufacturer’s protocol (Promega, Madison, WI, USA). A total of 200 pM TcdB were incubated with indicated concentrations of Dom in glucosylation buffer (50 mM HEPES, 100 mM KCl, 2 mM MgCl_2_, 1 mM MnCl_2_, 100 mg/L BSA, pH 7.5). TcdB substrate, recombinant Rac1 (5 µM), was added, and the reaction was started by addition of 100 µM UDP-glucose for 1 h at 37 °C. For control, the inhibitor of TcdB glucosyltransferase activity, castanospermin (10 mM, Santa Cruz Biotechnology), was included [50]. Three 10 µL aliquots of each sample were transferred to a white half area 96-well plate (Greiner Bio-One International GmbH, Kremsmünster, Austria) and treated with 10 µL of UDP detection reagent for 30 s on a plate shaker. Luminescence was measured after 15–60 min by Tecan infinite M1000 Pro plate reader (Tecan, Männsdorf, Switzerland) with 750 ms integration time.

### 4.7. In Vitro Glucosylation of Rac1 by TcdB

In this stage, 40 µg cell lysate was incubated with 50 ng TcdB in glucosylation buffer (50 mM HEPES, 100 mM KCl, 2 mM MgCl_2_, 1 mM MnCl_2_, 100 mg/L BSA, pH 7.5) in the presence of Dom or VER at indicated concentrations for 2 h at 37 °C. Samples were analyzed by SDS-PAGE and Western blotting. Non-glucosylated Rac1 was detected by a specific antibody (1:500, #61065, BD Biosciences, Franklin Lakes, NJ, USA). Hsp90 was detected to confirm equal loading. Signals were detected using the ECL system. Signals were densitometrically quantified using ImageJ. Non-glucosylated Rac1 signals were normalized to Hsp90 signals and then to untreated control samples.

### 4.8. Toxin Translocation Assay

The assay was performed as described earlier [51]. In brief, cells were pre-incubated with the respective inhibitor for 30 min followed by another 30 min incubation with medium containing the inhibitor plus BafA1. BafA1, an inhibitor of the vesicular ATPase, prevents the normal toxin uptake route via escape from endosomes. Subsequently, cells were incubated on ice with TcdB for 30 min. Then, cells were challenged with warm acidic medium (pH 3.8) for 10 min at 37 °C triggering toxin pore formation as well as translocation of the enzyme domain across the cytoplasmic membrane directly into the cell cytosol. For control, cells were treated with neutral medium (pH 7.5). Then, medium was exchanged for fresh BafA1-containing medium (pH 7.5). Morphological changes (cell rounding) were monitored and documented as a specific endpoint of intoxication.

### 4.9. Transepithelial Electrical Resistance (TEER) Measurements

CaCo-2 cells were seeded in 24-well culture inserts with 0.4 µm pore size (Corning Inc., Kennebunk ME, USA). Cells were grown for 3–5 days. After 30 min pre-incubation with the respective inhibitor, 100 pM TcdB were added, and cells were further incubated at 37 °C. Inhibitors were applied from the basolateral, and TcdB was applied from the apical side of the filter insert. For control, cells were left untreated or were treated with DMSO as solvent control for inhibitors. TEER was measured every hour with the EVOM2 apparatus using a STX2 electrode (World Precision Instruments Inc., Sarasota, FL, USA). Raw resistance data were normalized to 0 h time point.

### 4.10. Reproducibility of Experiments and Statistics

All experiments were performed independently at least 3 times. Number of replicates is given in the respective figures. Representative results are shown in the figures. Western blots were cropped for display reasons only. Data were statistically analyzed as stated in the figure legends (**** *p* < 0.0001, *** *p* < 0.001, ** *p* < 0.01, * *p* < 0.05, ns = not significant *p* > 0.05).

## Data Availability

The datasets generated and/or analyzed during the current study are either included in the study or available from the corresponding author on reasonable request.
